# A method for stabilising the XX karyotype in female mESC cultures

**DOI:** 10.1242/dev.200845

**Published:** 2022-11-28

**Authors:** Andrew Keniry, Natasha Jansz, Peter F. Hickey, Kelsey A. Breslin, Megan Iminitoff, Tamara Beck, Quentin Gouil, Matthew E. Ritchie, Marnie E. Blewitt

**Affiliations:** ^1^ The Walter and Eliza Hall Institute of Medical Research, 1G Royal Parade, Parkville, VIC 3052, Australia; ^2^ The Department of Medical Biology, University of Melbourne, Parkville, VIC 3010, Australia

**Keywords:** Cell culture, Embryonic stem cells, X chromosome inactivation

## Abstract

Female mouse embryonic stem cells (mESCs) present differently from male mESCs in several fundamental ways; however, complications with their *in vitro* culture have resulted in an under-representation of female mESCs in the literature. Recent studies show that the second X chromosome in female, and more specifically the transcriptional activity from both of these chromosomes due to absent X chromosome inactivation, sets female and male mESCs apart. To avoid this undesirable state, female mESCs in culture preferentially adopt an XO karyotype, with this adaption leading to loss of their unique properties in favour of a state that is near indistinguishable from male mESCs. If female pluripotency is to be studied effectively in this system, it is crucial that high-quality cultures of XX mESCs are available. Here, we report a method for better maintaining XX female mESCs in culture that also stabilises the male karyotype and makes study of female-specific pluripotency more feasible.

## Teaser

**Summary:** Exploiting the X-linked reporters of the Xmas mESC system to improve female mESC culture facilitates the study of karyotypically correct cells and thus female pluripotency.

## INTRODUCTION

Female and male pluripotent stem cells differ genetically, epigenetically and functionally (Schulz et al., 2014; Choi et al., 2017a,b; Yagi et al., 2017; Zvetkova et al., 2005; Ooi et al., 2010; Genolet et al., 2021; Song et al., 2019). However, owing to complications with *in vitro* culture of female mouse embryonic stem cells (mESCs), the vast majority of mESC research has been performed on male mESC lines. The first confirmed mESC line was male (Bradley et al., 1984). Subsequently, lines employed as workhorse cells, e.g. E14, R1, J1 and Bruce4, were also male (Hooper et al., 1987; Li et al., 1992; Nagy et al., 1993; Köntgen et al., 1993). This has limited our understanding of sex-specific pluripotency and has impeded the study of female-specific processes, including X chromosome inactivation (XCI): the dosage compensation mechanism in female mammals whereby one of the two X chromosomes becomes stably silenced for the life of the organism (Disteche and Berletch, 2015; Jegu et al., 2017; Gendrel and Heard, 2011; Brockdorff and Turner, 2015). Female mESCs present in a more naïve state of pluripotency than male mESCs, displaying increased expression of naïve pluripotency markers (Schulz et al., 2014; Genolet et al., 2021; Song et al., 2019) and global hypomethylation (Schulz et al., 2014; Choi et al., 2017a,b; Yagi et al., 2017; Zvetkova et al., 2005; Ooi et al., 2010; Habibi et al., 2013). Consistently, naïve female mESCs are comparatively slow to exit pluripotency upon differentiation (Schulz et al., 2014). Female mESCs are karyotypically unstable, with XO cells spontaneously arising, then rapidly dominating cultures (Choi et al., 2017b; Yagi et al., 2017; Zvetkova et al., 2005; Keniry et al., 2022). Use of defined media for mESC culture exacerbates the fragility of female mESCs, likely because this drives mESCs further towards a naïve state (Marks et al., 2012; Leitch et al., 2013; Silva et al., 2009; Ficz et al., 2013). Known as 2i/LIF (Ying et al., 2008; Silva et al., 2008), this media provides defined conditions for mESC culture, including inhibitors of glycogen synthase kinase 3β (GSK3β) and MEK/ERK signalling pathways, allowing culture without serum or feeder cells that promote more heterogeneous cell populations. Although challenging for female mESCs, the benefits of defined media are clear, offering simpler, more homogenous and reproducible cultures, while enabling study of naïve pluripotency *in vitro.* The use of 2i/LIF also greatly improves the efficiency of deriving mESC lines *de novo* (Czechanski et al., 2014), thereby expanding the possibilities for experimental design.

Female mESCs have the unique property of being transcriptionally active from both X chromosomes, a feature shared only with cells of the inner cell mass from which they are derived, induced pluripotent stem cells (iPSCs) and primordial germ cells (Monk and McLaren, 1981; Tam et al., 1994; Kratzer and Chapman, 1981). Lineage committed cells display dosage compensation by XCI. Upon differentiation, female mESCs undergo XCI; following this, hypomethylation and the XO karyotype are no longer features of female cells, with XCI seemingly having a stabilising effect (Schulz, 2017). Evidence suggests the two active X chromosomes cause female mESCs to behave differently from male mESCs, as pluripotent XO cells have similar transcriptomes, epigenomes and differentiation potential as XY cells (Schulz et al., 2014; Choi et al., 2017b; Zvetkova et al., 2005; Song et al., 2019; Pasque et al., 2018). The cause of these phenotypes is still being uncovered; however, suppression of the differentiation-promoting MAP kinase pathway is involved (Schulz et al., 2014), with heterozygous mutation of the X-linked *Dusp9* and *Klhl13* genes able to repress MAP kinase target gene expression and partly induce a male-like state in female cells (Choi et al., 2017a; Genolet et al., 2021).

The XO karyotype is preferential for female mESCs and quickly dominates cultures; however, as the XX karyotype defines female mESCs, it is crucial that female pluripotency is studied in cultures maintaining high ratios of XX cells. This is particularly important for XCI studies, where undetected XO cells may confound results. Several seminal studies have been performed in XX mESCs (Schulz et al., 2014; Choi et al., 2017a,b; Yagi et al., 2017; Zvetkova et al., 2005; Ooi et al., 2010; Genolet et al., 2021; Song et al., 2019; Habibi et al., 2013), but there is a large barrier to entry in studying these cells owing to a lack of protocols utilising 2i/LIF while retaining XX karyotype.

Here, we exploit the X-linked reporters of our previously published Xmas mESC system (Keniry et al., 2022), to develop an approach to maximise XX mESCs in 2i/LIF. Furthermore, we find this method stabilises the karyotype of male mESCs. Our protocol improves female mESC culture, facilitating the study of karyotypically correct cells and thus female pluripotency more generally.

## RESULTS

### A method for stabilising the XX karyotype in female mESC cultures

**Fig. 1.**
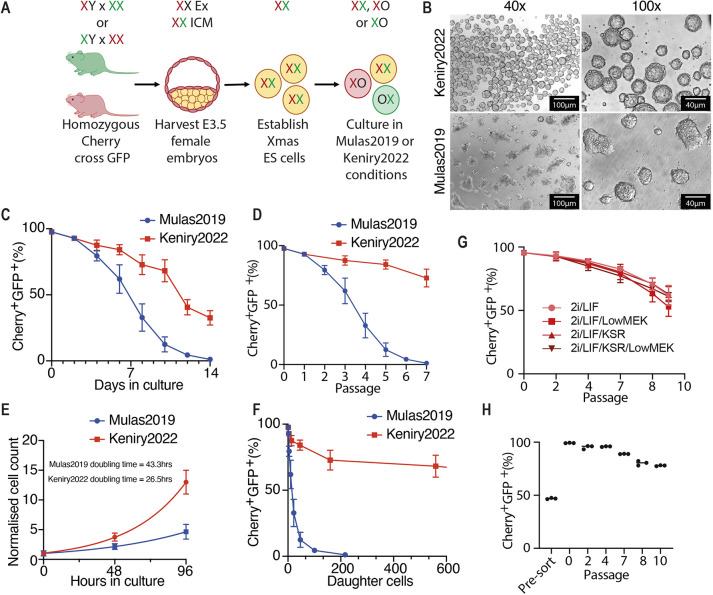
**Stabilisation of the XX karyotype in mESC culture.** (A) The Xmas mESC system identifies karyotype. (B) Bright-field images of Xmas mESCs grown under Keniry2022 and Mulas2019 conditions. (C) Flow cytometry data from Xmas mESCs maintained in 2i/LIF for 14 days in Keniry2022 and Mulas2019 conditions, where reporter alleles indicate karyotype. (D) Same data as in C transformed to reflect passage number. (E) Growth curves of Xmas mESCs under Keniry2022 and Mulas2019 conditions. Lines indicate non-linear fit (*n*=6). Doubling time calculations are given. (F) Same data as in C transformed by the doubling time of each method to reflect the number of XX daughter cells. (G) Flow cytometry data from Xmas mESCs maintained in Keniry2022 conditions with 2i/LIF supplemented with Knockout Serum Replacement (KSR) or reduced MEK inhibitor, or both (*n*=6). (H) Flow cytometry data from Xmas mESCs maintained in Keniry2022 conditions before and after fluorescence-activated cell sorting of GFP^+^mCherry^+^ cells (*n*=3). Data are mean±s.d.

We recently reported the Xmas mESC system, which carries X-linked mCherry and GFP reporter constructs driven by the hypoxanthine guanine phosphoribosyltransferase (*Hprt*) promoter *in trans* to each other, facilitating monitoring of XCI by flow cytometry with minimal manipulation of sensitive female mESCs (Keniry et al., 2022). As the X chromosome is biallelically expressed in mESCs, the Xmas reporter alleles also indicate the XX/XO karyotype ratio. Xmas reporter alleles are maintained as mouse lines, which when intercrossed produce female offspring with GFP and mCherry marking different X chromosomes (X*^Hprt^*^-GFP^ X*^Hprt^*^-mCherry^, Fig. 1A and Fig. S1A). This is important as it allows the constant rederivation of karyotypically normal primary Xmas mESC lines. Here, we exploit the Xmas system to optimise maintenance of the XX karyotype in culture. Starting with the current best practice 2i/LIF mESC culture method (Mulas et al., 2019), we iteratively and empirically determined features that stabilise the XX karyotype through hundreds of rounds of Xmas mESC derivations to arrive at an optimised protocol. Differences between our method (here termed Keniry2022) and the previously published approach (here termed Mulas2019; Mulas et al., 2019) include increased plating density, increased frequency of passaging (every 24 h), cells grown in suspension in non-tissue culture-treated plates and increased media volumes (Fig. 1B). All points of difference between protocols are detailed in Table 1, including our rationale for each change. A full protocol for our method is supplied in the supplementary Materials and Methods.

**Table 1.**
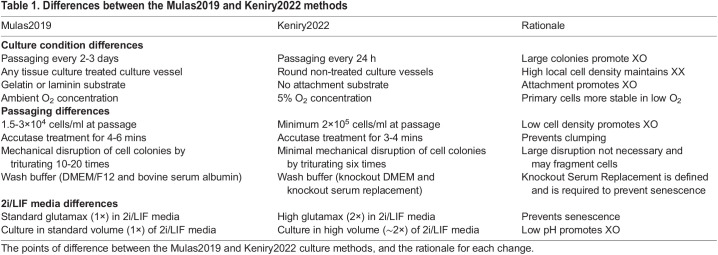
Differences between the Mulas2019 and Keniry2022 methods

Through these subtle changes to the existing best practice method, we substantially increase XX retention (Ying et al., 2008; Mulas et al., 2019), either when analysed as days in culture (Fig. 1C and Fig. S1B) or as passage number (p) (Fig. 1D). It takes 10-14 days after derivation to produce sufficient mESCs for these experiments; therefore, the XX karyotype is maintained until ∼20-24 days in culture. Moreover, our method reduces doubling time for XX cells (26.5 h for Keniry2022 versus 43.3 h for Mulas2019; Fig. 1E), meaning we produce substantially more XX daughter cells per derivation (Fig. 1F). Previous studies have improved female mESC culture, achieving stabilisation of the epigenome, but not XX karyotype (Choi, 2017a,b; Yagi et al., 2017). Despite improvement, we were unable to prevent XO cells becoming predominant in cultures; however, our method allows XX lines to be expanded to quantities sufficient for most experimental procedures, as we recently demonstrated (Keniry et al., 2022).

With the method optimised, we tested whether simple alterations to 2i/LIF improved karyotype retention. Low concentration of MEK inhibitor reportedly improves the genomic stability of mESCs (Choiet al., 2017b; Yagi et al., 2017; Di Stefano et al., 2018); however, halving the concentration had no effect on XX karyotype retention in Xmas mESCs (Fig. 1G). We also supplemented 2i/LIF with 2% knockout serum replacement, again finding no improvement, both in normal and low-MEK inhibitor conditions. Finally, we tested whether XX Xmas mESCs could be enriched by sorting mCherry and GFP double-positive cells by fluorescence-activated cell sorting (FACS), finding we could purify lines with a high proportion of XX mESCs. After sorting, these lines became XO at a rate similar to unsorted cells, suggesting that this may be a viable strategy for XX retention in Xmas mESCs (compare Fig. 1H with Fig. 1C,D).

### The modified method stabilises the XX karyotype of F1 mESC lines

**Fig. 2.**
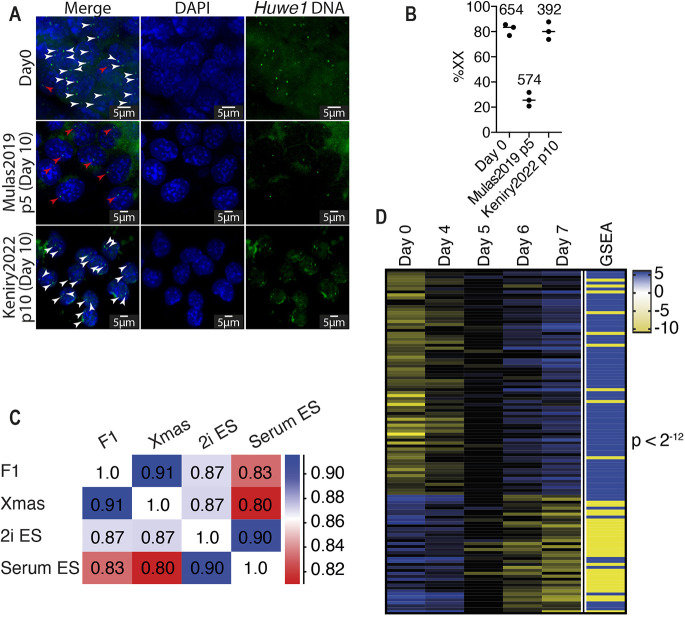
**The XX karyotype is stabilised in multiple strain backgrounds.** (A) DNA fluorescent *in situ* hybridisation for the *Huwe1* region of chromosome X in X^FVB^X^CAST^ mESCs cultured in Mulas2019 conditions for five passages (p5, 10 days) or Keniry2022 conditions for ten passages (p10, 10 days). White and red arrowheads mark *Huwe1* DNA in XX and XO cells, respectively. (B) Quantification of data from A. Numbers indicate cells counted. (C) Pearson correlation of our published RNA-seq from X^FVB^X^CAST^ and Xmas mESCs cultured in Keniry2022 conditions with published RNA-seq from mESCs grown in serum or 2i/LIF (Marks et al., 2012; Leitch et al., 2013). (D) Expression values (rpm log_2_) from our X^FVB^X^CAST^ RNA-seq during differentiation compared with a mESC differentiation gene set (GSEA). Yellow indicates downregulation and blue indicates upregulation in the GSEA gene set. *P*-value determined by Chi-square test.

We developed our method using Xmas mESCs that are from a C57Bl/6 strain background. To test whether the method benefits another background, we derived mESCs from FVB/NJ (FVB)/CAST/EiJ (CAST) F1 blastocysts and, after an initial 10-14 day growth period in Keniry2022 conditions, we split individual lines and passaged them for 10 days in Keniry2022 or Mulas2019 conditions to compare the outcomes. As these lines lack Xmas reporters, we measured the X karyotype by DNA florescence *in situ* hybridisation (FISH) with a BAC against the *Huwe1* region of chromosome X. We found substantial stabilisation of the XX karyotype when cells were cultured by our method (Fig. 2A,B), suggesting it improves XX retention in mESC lines of diverse strain backgrounds.

### The modified method does not alter mESC identity

We next sought to identify potential effects of our conditions on cell identity, by comparing published mESC RNA-seq from Xmas and FVB×CAST F1 mESCs grown under our conditions, with mESCs grown in 2i/LIF or serum-containing media under traditional conditions (Keniry et al., 2022; Marks et al., 2012; Maza et al., 2015). All datasets were highly correlated; however, cells grown using our method were more highly correlated with mESCs grown in 2i/LIF than in serum-containing media (Fig. 2C and Fig. S1C). This was to be expected, as our conditions use 2i/LIF. Therefore, the naïve pluripotent state expected in 2i/LIF mESCs is likely maintained by our modified method.

We next sought to determine the differentiation potential of mESCs cultured under our conditions by reanalysing our published RNA-seq for FVB×CAST F1 mESCs during differentiation (Keniry et al., 2022). We compared these data with a mESC differentiation gene set and found very high correlation (*P*<2^−12^, Fig. 2D), suggesting that mESCs under our conditions differentiate with similar transcriptional kinetics to known mESCs. We have previously shown that Xmas mESCs under our conditions form teratomas containing differentiated cells of all three germ layers upon injection into nude mice (Keniry et al., 2022). Importantly, XCI proceeds with the expected epigenetic hallmarks when mESCs grown under our modified conditions are differentiated *in vitro* (Keniry et al., 2022).

### The modified culture conditions maintain DNA hypomethylation of XX cells

**Fig. 3.**
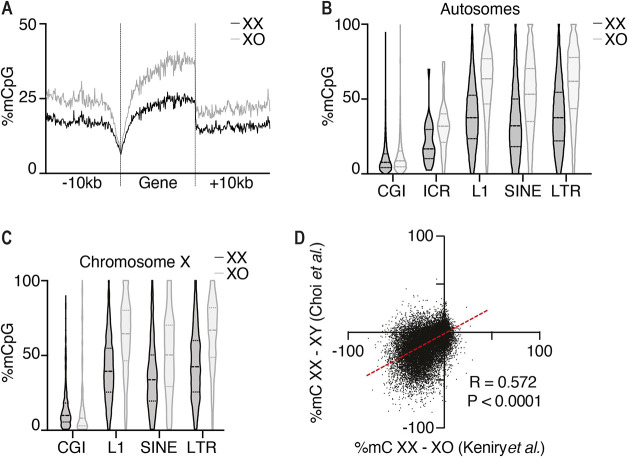
**The modified culture conditions maintain female mESC hypomethylation.** RRBS in FACS XX and XO Xmas cells cultured by our modified method for 12 passages. (A) Average CpG methylation across all autosomal genes ±10 kb. (B,C) CpG methylation at CpG islands (CGIs), imprinting control regions (ICRs) and repetitive elements (L1, SINE and LTR). Dashed line indicates median; dotted lines indicate 25th and 75th centiles. Autosomal (B) and X-linked (C) features are shown separately. (D) Comparison of RRBS with published RRBS (Choi et al., 2017a). Dots represent informative CpGs, dashed red line indicates average. Pearson correlation R-value and associated *P*-values are given.

DNA methylation has a stabilising effect on the genome; therefore, we tested whether our conditions maintain karyotype by relieving XX-associated hypomethylation. We grew Xmas mESCs under our conditions and separated XX and XO populations by FACS of the Xmas reporter alleles, then measured DNA methylation by reduced representation bisulfite sequencing (RRBS). This revealed XX mESCs remain globally hypomethylated compared with XO cells (Fig. 3A), including at imprinting control regions and repetitive elements (Fig. 3B,C). There was no difference at CpG islands, which are already hypomethylated (Fig. 3B,C). This trend was observed at both autosomal and X-linked loci. These data suggest the stabilised XX karyotype achieved by our conditions is not due to increased DNA methylation. Therefore, the hypomethylated epigenome of XX mESCs is maintained by our method, suggesting these cells are a suitable model for studying this property of female pluripotency. Conversely, our method is unlikely to stop erosion of DNA methylation observed at imprinting control regions in females (Choi et al., 2017b; Yagi et al., 2017).

To determine whether the differential methylation observed between XX and XO Xmas mESCs occurs at CpG sites that are common to differential methylation between female and male mESCs, we reanalysed published RRBS data for XX and XY mESCs (Choi et al., 2017a). As stated in the publication, XX mESCs were hypomethylated compared with XY mESCs, with the sites of hypomethylation being highly correlated with our dataset, suggesting similar loci are differentially methylated between male and female mESCs as for XX and XO mESCs cultured by our method (Fig. 3D).

### The modified culture system improves male mESC fitness

**Fig. 4.**
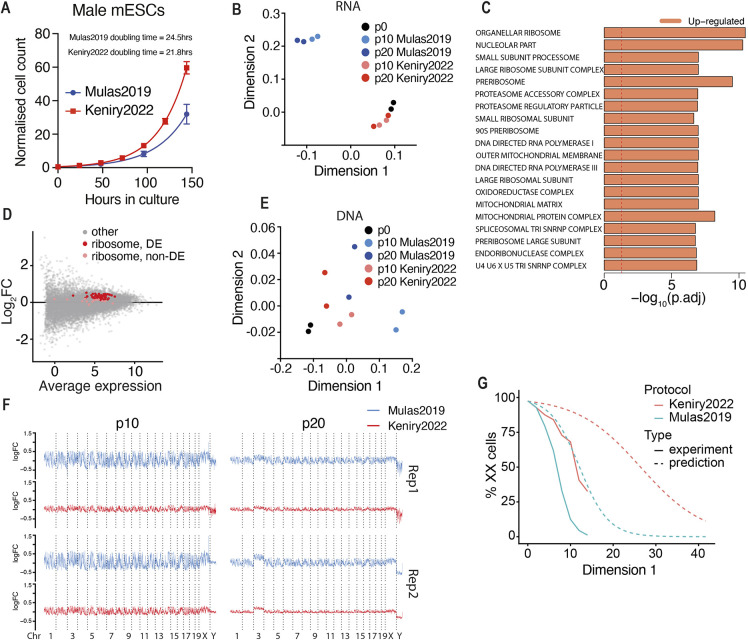
**The modified culture conditions maintain the transcriptome and karyotype of male mESCs.** (A) Growth curves of male mESCs in Mulas2019 or Keniry2022 conditions. Lines indicate non-linear fit (*n*=3). Data are mean±s.d. Calculated doubling times are given. (B) Multi-dimensional scaling plot of RNA-seq data from p0 male mESCs and cells in Mulas2019 or Keniry2022 conditions at p10 and p20 (*n*=2). Mulas2019 has 48 h passaging compared with 24 h in Keniry2022. To compare days in culture, rather than passage, p10 of Mulas2019 and p20 of Keniry2022 are both 20 days culture beyond p0. (C) Gene set testing of p10 and p20 samples in Mulas2019 or Keniry2022 conditions. Dashed line indicates *P*=0.05. (D) MA plot showing fold change (log_2_FC) at genes in male mESCs grown in Mulas2019 or Keniry2022 conditions. Significantly and non-significantly differentially expressed ribosomal genes are indicated in red and pink, respectively. (E) MDS plot of DNA-seq in 1 Mb bins from p0 male mESCs compared with cells cultured in Mulas2019 or Keniry2022 conditions at p10 and p20 (*n*=2). (F) Chromosome coverage plots for male mESCs cultured in Mulas2019 or Keniry2022 conditions. Reads normalised to equivalent positions in p0 samples (*n*=2). (G) Modelling of XX/XO mESC populations grown under Mulas2019 or Keniry2022 conditions based on experimentally determined doubling times. Experimentally determined loss of XX cells, from Fig. 1C, is shown for comparison.

We next tested our method for the culture of more commonly used male mESCs, finding our method decreases doubling time of male mESCs (21.8 h for Keniry2022 versus 24.5 h for Mulas2019; Fig. 4A), although the scale of effect was less than in females.

To delve further into how our method may affect male mESCs, we performed a series of genomic experiments after culture with either our protocol or the Mulas2019 protocol. We present these data as passage matched, which most closely aligns the number of cell divisions for each method; however, for comparison of days in culture p20 by the Keniry2022 method is analogous to p10 of the Mulas2019 method. Principal component analysis of RNA-seq showed that our protocol maintained male mESCs transcriptionally similar to cells freshly derived from blastocysts, whereas cells under Mulas2019 conditions diverged (Fig. 4B and Fig. S2A)*.* We identified 5526 differentially expressed genes between methods, with gene set testing revealing significant upregulation of ribosome and mitochondrial genes in cells maintained under Keniry2022 conditions (Fig. 4C,D and Table S1), consistent with the more rapid self-renewal we observe.

DNA-seq on these cells revealed no major karyotypic abnormalities on a chromosome-wide scale (Fig. S2B). However, Y chromosome loss was observed in one line, occurring during derivation, before our experimental culture conditions (Fig. S2B). We are unsure why this occurred; however, Y chromosome instability in culture has been reported (Xu et al., 2017; Liang et al., 2008). Despite no chromosome-wide differences, principal component analysis revealed autosomes from cells cultured under our conditions remained most similar to those of freshly derived cells, whereas cells cultured using the Mulas2019 method diverged (Fig. 4E); consistent with our method stabilising karyotype. Analysing the genome in 1 Mb bins revealed no differentially represented regions in cells cultured using our method (FDR<0.05, log_2_FC>1.1). By contrast, the Mulas2019 method had ∼57% of the genome differentially represented at p10 and ∼5.5% at p20, suggesting prolonged culture selects against major karyotypic abnormalities (Fig. 4F and Table S2). Even when considering equivalent time in culture (p10 Mulas2019 versus p20 Keniry2022), our protocol markedly improves karyotype maintenance. Therefore, our method improves karyotypic maintenance of both male and female mESCs.

As our protocol was optimised empirically, we cannot be sure why it better preserves karyotype or reduces doubling time, but we believe it is due to decreased cell stress. Although our method shortened doubling time of both male and female mESCs, the improvement was greater in XX (∼39% shorter, Fig. 1E) compared with XY (∼11% shorter) mESCs, suggesting cells under more stress benefit most from our method. This provides an explanation of why our method maintains the XX karyotype longer, as an unequal decrease in doubling time between XX and XO cells would lead to XO cells taking longer to out compete and dominate cultures. Indeed, mathematical modelling of this scenario, using the doubling times for XX and XY mESCs, suggests this is more than sufficient to explain the increased XX retention we observe (Fig. 4G). That this model overestimates observed XX retention suggests factors not included in our model, such as a high rate of spontaneous XO conversion, contribute to XX retention. A previous publication reports mESC doubling times between 12 and 30 h (Tamm et al., 2013) in male lines tested. Another study directly compared growth kinetics of male and female mESCs (Song et al., 2019). Similarly, XY cells propagated faster than XX mESCs; however, female cells doubled faster than we observed (every 16.5 h). We are unsure why these cells grow faster; however, the use of F1 hybrid mESCs and serum-containing media is likely to contribute.

Female mESCs in serum/LIF have been used successfully in XCI studies for many years; although they require regular karyotype monitoring, it seems that specific lines maintain a more constant XX to XO ratio than mESCs in 2i/LIF. Cells in serum/LIF media are subject to pro-differentiation signals, but activation of JAK-STAT2 signalling by LIF prevents them from fully differentiating; rather, they maintain a heterogeneous state of primed pluripotency (Niwa et al., 1998; Matsuda, 1999). This may reduce pressure to lose an X chromosome in some cells, eventually stabilising the XX/XO ratio. There are many benefits to using defined 2i/LIF to maintain mESCs in a naïve state, including ease of culture, consistency between experiments, homogeneous cell populations and ease of derivation. In some cases, studying establishment of XCI from the naïve state, as opposed to primed, may be appropriate, depending on the question. Therefore, robust protocols that maintain XX mESCs in defined media are beneficial.

As XO female mESCs lose the unique properties of female pluripotency, it is crucial to maintain high quality cultures of XX mESCs for research. Here, we provide a protocol that makes this more achievable, allowing further discovery of previously hidden facets of this unique female cell type.

## MATERIALS AND METHODS

### Animal strains and husbandry

Animals were housed and treated according to the Walter and Eliza Hall Institute (WEHI) Animal Ethics Committee approved protocols (2014.034, 2018.004, 2020.050 and 2020.047). Xmas mice are on a C57BL/6 background and have been published previously (Keniry et al., 2022). Castaneus (CAST/EiJ) mice were obtained from Jackson laboratories and are maintained at WEHI. FVB/NJ mice were obtained from stocks held at WEHI. Oligonucleotides used for genotyping have been previously published (Keniry et al., 2022).

### Derivation of mESCs

Mouse ESCs were derived as previously described (Keniry et al., 2022). For further details, see the supplementary Materials and Methods.

### Modified culture method for mESCs (Keniry2022)

We provide a fully detailed lab protocol in the supplementary Materials and Methods. In brief, mESCs were maintained in 2i/LIF medium (Ying et al., 2008) [KnockOut DMEM (Life Technologies), 1× Glutamax (Life Technologies), 1× MEM Non-Essential Amino Acids (Life Technologies), 1×N2 Supplement (Life Technologies), 1×B27 Supplement (Life Technologies), 1× β-mercaptoethanol (Life Technologies), 100 U/ml Penicillin/100 µg/ml Streptomycin (Life Technologies), 10 µg/ml Piperacillin (Sigma-Aldrich), 10 µg/ml Ciprofloxacin (Sigma-Aldrich), 25 µg/ml Fluconazol (Selleckchem), 1000 U/ml ESGRO Leukemia Inhibitory Factor (Merck), 1 µM StemMACS PD0325901 (Miltenyi Biotech) and 3 µM StemMACS CHIR99021 (Mitenyi Biotech)] in suspension culture on non-tissue culture treated plates at 37°C in a humidified atmosphere with 5% (v/v) carbon dioxide and 5% (v/v) oxygen. Antibiotic and antimycotics are added to prevent contamination from flushing during the derivation process. We also use versions of 2i/LIF media that contain either low MEK inhibitor (0.5 µM StemMACS PD0325901) or knockout serum replacement (2% final concentration, Life Technologies). Daily passaging of mESCs was performed by allowing unattached colonies to settle in a tube for less than 5 min. Supernatant containing cellular debris was removed before resuspension in Accutase (Sigma-Aldrich) and dissociation at 37°C for 5 min to achieve a single-cell suspension. At least four volumes of mESC wash media [KnockOut DMEM (Life Technologies), 10% KnockOut Serum Replacement (Life Technologies), 100 IU/ml penicillin/100 µg/ml streptomycin (Life Technologies)] were added to the suspension and cells were pelleted by centrifugation at 600 ***g*** for 5 min and plated into an appropriately sized non-tissue culture-treated plate, never flasks, in an excess of 2i/LIF media. Cells were assessed for XX karyotype regularly by flow cytometry.

For comparison of our method with that of Mulas et al. (2019), Xmas mESC lines were derived over approximately 10 to 14 days. At the beginning of our experimental timecourse, each line was split into both culture conditions, such that each replicate has a matched sample in each condition.

We also used a variant of this method where cells were grown on plates coated with 0.1% gelatin when producing Fig. S1B. Culture conditions were otherwise identical.

### Current best practice culture method for mESCs (Mulas2019)

For comparison of our method, we used the current state-of-the-art method for mESC culture (Mulas et al., 2019), exactly as described apart from the addition of Piperacillin, Ciprofloxacin and Fluconazol to the 2i/LIF media, as described above. Aside from this, all reagents were as for our modified culture method. The primary differences of the Mulas2019 culture method are that plating density is decreased, passaging occurs every 48 h, cells grow on a gelatin substrate in tissue culture-treated plates and media volumes are decreased compared with the Keniry2022 method.

### Differentiation of mESCs

Our mESC differentiation protocol has been published previously (Keniry et al., 2022). Briefly, we employ undirected differentiation by transitioning cells from 2i/LIF media into DME HiHi media (DMEM, 500 mg/l glucose, 4 mM L-glutamine, 110 mg/l sodium pyruvate, 15% fetal bovine serum, 100 U/ml penicillin, 100 μg/ml streptomycin, 0.1 mM nonessential amino acids and 50 μM β-mercaptoethanol) in 25% increments every 24 h. Cells continue to differentiate in 100% DME HiHi media. For staging, we refer to the first day of differentiation as being day 0, when cells are placed into 75% 2i/LIF and 25% DME HiHi.

### Flow cytometry analysis and sorting

Cells were prepared in KDS-BSS with 2% (v/v) FBS, with dead cells and doublets excluded by size and analysed using a BD LSRFortessa cell analyser. Flow cytometry data were analysed using FlowJo. For fluorescence-activated cell sorting, single cell suspensions were prepared in KDS-BSS buffer with 10% Knockout Serum Replacement (KSR, Life Technologies). Sorting was performed with a BD FACSAria Fusion, with cells collected in 100% KSR.

### RNA-seq library generation and analysis

For the RNA-seq depicted in Fig. 2C,D, we compared published datasets (Marks et al., 2012; Maza et al., 2015). For the RNA-seq depicted in Fig. 4B-D, we derived male C57Bl/6 mESCs using our culture methods, for two independent lines. These cells were then split in two (p0) and cultured for ten and 20 passages using either the conditions provided in this report or the Mulas2019 method. Cells were collected by the addition of lysis buffer and RNA was extracted using the Quick-RNA MiniPrep kit (Zymo Research). Next Generation Sequencing libraries were prepared using by TruSeq RNA sample preparation kit (Illumina). Samples were sequenced in-house on the Illumina NextSeq500 platform producing 75 bp single-end reads. Quality control and adapter trimming were performed with fastqc and trim_galore (http://www.bioinformatics.babraham.ac.uk/projects/trim_galore), respectively. Reads were aligned to the mm10 reference genome using histat2 (Kim et al., 2015). Expression values in reads per million (RPM) were determined using the Seqmonk package (www.bioinformatics.babraham.ac.uk/projects/seqmonk/), using the RNA-seq Quantitation Pipeline. Initial data interrogation was performed using Seqmonk.

Gene set testing and differential gene expression analysis were performed by making two groups by pooling samples at all passages from either the Mulas2019 culture method or the Keniry2022 method. Genes with expression values below 0 rpm log_2_ were filtered from the analysis. Differential expression analysis between the two mESC culture methods was performed on gene-level counts with TMM normalisation, filtering out genes expressed in fewer than half the samples, using edgeR v3.26.7 (Robinson et al., 2010; McCarthy et al., 2012). Model-fitting was performed with voom v3.40.6 (Law et al., 2014) and linear modelling followed by empirical Bayes moderation using default settings. Differential expression results from voom were used for gene set testing with EGSEA v1.12.0 (Alhamdoosh et al., 2017) against the c5 Gene Ontology annotation retrieved from MSigDB, aggregating the results of all base methods by ‘fry’ and sorting by median rank.

### DNA-seq library preparation and analysis

We derived male C57Bl/6 mESCs using our culture methods for two independent lines. After an 18 day derivation period, cells were split in two (P0) and cultured for ten and 20 passages using either the Keniry2022 conditions given in this report or the Mulas2019 method, described above and by Mulas et al. (2019). Note that the Mulas2019 method has 48 h splitting rather than the 24 h splitting used in the Keniry2022 method; thus, to compare samples that are days in culture matched, rather than passage matched, p10 of Mulas2019 and p20 Keniry2022 are both 20 days in culture beyond p0. Sequencing libraries were prepared using the TruSeq DNA sample preparation kit (Illumina) and sequenced in-house on the Illumina NextSeq500 platform with 75 bp single-end reads. Reads were mapped to mm10 with bowtie2 (Langmead and Salzberg, 2012) and counted in 1 Mb bins along the genome using the GenomicAlignments R/Bioconductor package (Lawrence et al., 2013). The percentage of reads mapped to each chromosome was calculated. Only bins on the autosomes and sex chromosomes were included and those bins overlapping the ENCODE blacklisted regions were excluded. For each sample, we computed the coverage of each bin in log counts per million. We then computed the log fold changes by comparing each sample with the relevant p0 sample and plotted these by bin position along the genome. We used the edgeR R/Bioconductor package (Robinson et al., 2010) to perform a multidimensional scaling plot of distances between samples based on the log fold changes. Differential abundance analysis was performed using edgeR and limma (Ritchie et al., 2015). Briefly, the voom method (Law et al., 2014) was used to prepare count data for linear modelling and the within-cell-line correlation estimated using the ‘duplicateCorrelation’ function from the limma package (Smyth et al., 2005). The voom method was then re-applied (now accounting for the within-cell-line correlation), the within-cell-line correlation was re-estimated and these transformed data were used as the input to a linear model with a design matrix encoding the passage number and protocol of each sample, while blocking the cell line and including the estimated within-cell-line correlation when fitting the linear models. We used the empirical Bayes statistics (Phipson et al., 2016) to test for differential abundance at p10 versus p0 and p20 versus p0 within each protocol at a false discovery rate of 0.05 and requiring a minimum log2-fold change of 1.1 (McCarthy and Smyth, 2009).

### DNA fluorescence *in situ* hybridisation

DNA fluorescence *in situ* hybridisation (FISH) was performed as previously described (Chaumeil et al., 2008) on female mESCs derived by crossing FVB/NJ (FVB) dams with CAST/EiJ (CAST) sires, then passaged with either the Mulas2019 or the Keniry2022 method. The X chromosome was detected with a BAC probe against the *Huwe1* region (RP24-157H12), as previously described (Patrat et al., 2009). The probe was labelled with Green-dUTP (02N32-050, Abbott) using nick translation (07J00-001, Abbott). The cells were mounted in Vectashield antifade mounting medium (Vector Laboratories) and visualised on LSM 880 or LSM 980 microscopes (Zeiss). Images were analysed using the open source software FIJI (Schindelin et al., 2012).

### Reduced representation bisulfite sequencing

Xmas mESCs were cultured for 12 passages using our modified method, before being FACS separated into XX and XO populations based on the Xmas reporter alleles, using a BD FACSAria III Cell Sorter. Reduced representation bisulfite libraries were prepared and analysed as previously described (Yin et al., 2016). For the comparison with published data shown in Fig. 4D, we used data from Choi et al. (2017a) (a GEO deposit with accession number GSE68733).

### Growth curve modelling

We modelled the proportion of XO cells as a function of time in culture as 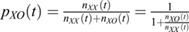, with the numbers of XX and XO cells following simple exponential growths with doubling frequencies *λ*_*XX*_ and *λ*_*XO*_:

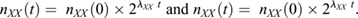


Thus,

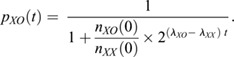


Based on the experimental curves, for the Mulas2019 and Keniry2022 protocols,

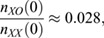
while the division frequencies derived from Fig. 1E gave: Δ*λ*_*Mulas*_=*λ*_*XO*_−*λ*_*XX*_=(24.5)^−1^−(43.3)^−1^=0.0177 *h*^−1^ and Δ*λ*_*Keniry*_=(21.8)^−1^−(26.5)^−1^=0.00814 *h*^−1^.

Plugging these numbers into the formula for the proportion of XO cells illustrated that a smaller difference in division frequencies (Δλ) under the new conditions could easily explain the longer retention of the XX karyotype. Data were plotted in R 4.2.1 with the ggplot and cowplot packages.

### Statistics

Sample sizes (*n*) are given in the figure legends. As effect sizes were unknown before experiments, no power calculations were performed. No data were excluded. Biological replicates were typically mESC lines derived from different individual embryos, with test and control experiments performed on the same mESC line. Experiments were not blinded or randomised. Experiment-specific statistical tests are detailed in the figure legend for that experiment. All tests were two-tailed and multiple testing corrections were applied as appropriate.

## Supplementary Material

10.1242/develop.200845_sup1 Supplementary information
