# Supplementary material for: A method for stabilising the XX karyotype in female mESC cultures
Source: Development. 2022 Nov 28;149(22):dev200845. doi: 10.1242/dev.200845 (PMC10112917; doi:10.1242/dev.200845)
Supplement: Supplementary file 1 [file develop-149-200845_review_history.pdf]

A method for stabilising the XX karyotype in female mESC cultures

Andrew Keniry, Natasha Jansz, Peter F. Hickey, Kelsey A. Breslin, Megan Iminitoff, Tamara Beck, Quentin Gouil, Matthew E. Ritchie and Marnie E. Blewitt

DOI: 10.1242/dev.200845

Editor: Haruhiko Koseki

Review timeline

Original submission:	11 April 2022
Editorial decision:	31 May 2022
First revision received:	9 September 2022
Editorial decision:	19 October 2022
Second revision received:	25 October 2022
Accepted:	30 October 2022

Original submission

First decision letter

MS ID#: DEVELOP/2022/200845

MS TITLE: A method for stabilising the XX karyotype in female mESC cultures

AUTHORS: Andrew Keniry, Natasha Jansz, Peter F Hickey, Kelsey Breslin, Megan Iminitoff, Tamara Beck, Matthew E Ritchie, and Marnie E Blewitt

I have now received all the referees' reports on the above manuscript, and have reached a decision. The referees' comments are appended below, or you can access them online: please go to BenchPress and click on the 'Manuscripts with Decisions' queue in the Author Area.

As you will see, the referee 2 and 3 express considerable interest in your work and find potential contribution of your work to the X chromosome biology field. However, they, together with reviewer 1, have some significant criticisms and recommend a substantial revision of your manuscript before we can consider publication. If you are able to revise the manuscript along the lines suggested, which may involve further experiments, I will be happy receive a revised version of the manuscript. Your revised paper will be re-reviewed by one or more of the original referees, and acceptance of your manuscript will depend on your addressing satisfactorily the reviewers' major concerns. Please also note that Development will normally permit only one round of major revision. If it would be helpful, you are welcome to contact us to discuss your revision in greater detail. Please send us a point-by-point response indicating your plans for addressing the referee’s comments, and we will look over this and provide further guidance.

Please attend to all of the reviewers' comments and ensure that you clearly highlight all changes made in the revised manuscript. Please avoid using 'Tracked changes' in Word files as these are lost in PDF conversion. I should be grateful if you would also provide a point-by-point response detailing how you have dealt with the points raised by the reviewers in the 'Response to Reviewers' box. If you do not agree with any of their criticisms or suggestions please explain clearly why this is so.

© 2022. Published by The Company of Biologists under the terms of the Creative Commons Attribution License (https://creativecommons.org/licenses/by/4.0/).

Reviewer 1

Advance summary and potential significance to field

In this manuscript, Keniry et al. describe a new protocol to allow XX mouse ES cells to maintain their XX karyotype in a long-term culture compared to the 2i/LIF condition, which has been used as the best protocol to maintain naïve mouse ES cells. XX ES cells are known to easily lose one X chromosome during culture and become X0 karyotype. The development of an optimized culture condition to maintain XX karyotype is beneficial to investigate XCI as well as the pluripotency of female cells.
By modifying the 2i/LIF culture condition, the paper shows XX ES cells can retain their XX karyotype as long as 10 days. These female ES cells also have a genome-wide CpG hypomethylation, another feature of the pluripotency of XX ESCs. The paper also shows that male ESCs can stably retain transcriptome and karyotype in this new culture condition.
Overall, the paper defines a novel culture condition that allows XX ES cells to be maintained in the naïve pluripotency for longer time than 2i/LIF. Female XX ES cells, however, retain XX karyotype until day 10 and then appear to start a rapid loss of one X chromosome with the new method. The XX karyotype is maintained about 2 times longer with the new method than the conventional 2i/LIF condition (10 days vs 5 days based on Fig. 1B), which would not be a strong improvement. In addition, the authors also describe that FACS sorting to select XX GFP/mCherry double-positive cells does not work effectively, narrowing the possibility to maintain XX karyotype. I, therefore, think that the current manuscript does not have much improvement and cannot be accepted for publication without adding more strong data.

Comments for the author

Major points:
At least three major points need to be addressed and added to the current manuscript.
1. It is critical to extend the duration of XX karyotype retention. The current data shows XX karyotypes can be maintained until day 10, which would not be long enough for several experiments such as creating a stable transgenic line. Therefore, I think XX karyotype should be maintained for a longer time.
2. Alternatively, even if it is difficult to maintain XX karyotype for more than 10 days, a method to select XX cells from X0/XX mixed population would overcome this limitation. This point needs to be further examined.
3. Do these ES cells cultured with the new method have more efficient germ line transmission? The efficiencies of germ line transmission with both male and female ES cells cultured with the new method should be examined. As male ES cells retain transcriptome and karyotype, this new method would also improve the germ line transmission of male ES cells than 2i/LIF condition. It is also important to examine the efficiency of germ line transmission of female ES cells to study the female pluripotency.

Specific points:
1. In Fig. 2C, the authors describe that the XX FVB/CAST transcriptome obtained with the new culture condition was more highly correlated with the one obtained with 2i/LIF culture condition than serum-containing medium. In Fig. 4A, however, they mention that male ES cells cultured with the new protocol and 2i/LIF have different PCA profiles. These descriptions are quite confusing. Is it possible for the authors to perform a pearson correlation analysis for male ES cells and PCA for female ES cells in both the new culture condition and 2i/LIF?
2. They show that female ES cells have longer doubling time, 26.5 hr with the new culture condition and 43.4 hr with 2i/LIF. They also describe that male ES cells with 2I/LIF grow more rapidly (12 – 30 hr). Is this shorter doubling time specific for female ES cells? How fast do male ES cells grow in the new culture condition?
3. In Fig. 2A, the authors perform DNA FISH with Huwe1 probe and show that the majority of XX ES cells retain two Huwe1 spots. However, two Huwe1 spots could also represent X0 tetraploid karyotype? To avoid this possibility, it is necessary to perform flowcytometry or DNA FISH with an autosomal probe to prove that they are diploid, but not tetraploid.
4. They describe that lower concentration of MEK inhibitor does not solve the problem entirely. The authors should clearly mention which problem they were not able to solve.

© 2022. Published by The Company of Biologists under the terms of the Creative Commons Attribution License (https://creativecommons.org/licenses/by/4.0/).

5. Although it is briefly described in the text, it is still difficult to understand which points the authors have modified from the 2i/LIF protocol. I think the authors should present a table that shows the differences between the new protocol and the prior 2i/LIF condition. In addition, it is helpful to show the pictures of ES cells cultured in the new condition. Many researchers may not be familiar with suspension culture of ES cells and need to see how the cells look like.
6. In Fig. 3, the authors show that XX ES cells grown with the new method retain hypomethylated CpG in repeat elements. The passage number of ES cells used in this analysis needs to be indicated.

Reviewer 2

Advance summary and potential significance to field

In this manuscript the authors introduce a new method to culture mouse ESCs which stabilizes against the loss of an X chromosome in XX females. They further characterize the genome, transcriptome and methylome of cells grown in their culture conditions and previously published conditions, demonstrating that their culture conditions more closely resemble freshly derived ESC cultures. We think that this will be a valuable contribution to the field, as X chromosome loss is very problematic for studying X chromosome inactivation and X chromosome content should be considered in all studies to ensure results are relevant to both males and females. See comments below for how we believe this manuscript could be improved:

Comments for the author

For your RRBS, you only compare your XX and XO populations. Could you compare these to a male or freshly derived female RRBS sample to demonstrate that the hypomethylation seen in your XX cells is proper and that it is the XO cells with problematic DNA methylation.

In your RRBS, could you separately compare X chromosome DNA methylation and autosomal DNA methylation, to see if X chromosome differences may be driving the hypomethylation differences you observe.

2nd paragraph page 6, I think there is a typo saying Keniry2019. I think it should instead be Keniry 2022 or Mulas 2019

I suggest the addition of a small table demonstrating the difference between your culture conditions and those of Mulas2019, as this may provide an easily digestible summary of what you have changed.

Figure 2B: Please provide the number of cells counted per condition.
In the protocol I did not understand this sentence “Note, when passaging low numbers of cells the last drop should be blotted with a tissue to avoid significantly diluting the 2i media.“
Can you provide the brand and catalogue number of the “round wells of non-tissue culture treated plates "
Can you discuss the doubling times of Song et al Stem Cell Reports 2019?
Can you describe better, more specifically, the new changes to the protocol in the main manuscript on page 4?

Reviewer 3

Advance summary and potential significance to field

Keniry et al. using their recently-derived double-reporter female cell line and other mouse ES lines, report the set-up of a new protocol to stabilize the XX genotype in mouse female ESCs during culturing. Working with female ESCs is hard, and this protocol might bring some relief/help to people working in X Chromosome Inactivation (XCI). It might also encourage more widespread use of female ESCs in research. Therefore I think that this work is important. Not just for the XCI field but for the whole stem cell community. The manuscript, however, can be further improved. If my criticisms are properly addressed, this work can be accepted for publication.

© 2022. Published by The Company of Biologists under the terms of the Creative Commons Attribution License (https://creativecommons.org/licenses/by/4.0/).

Comments for the author

Major criticism:
1. Considering how many variables can be optimized in such types of protocol, it is not clear - at all - how you arrived at the final version of your improved protocol. What was the primary rationale for setting up different culturing conditions or handling procedures such as splitting? How many conditions were tested? Can you exclude that the genetics/epigenetics of the cell lines used in this work contributed to the observed phenotype?

1. There is a lack of rationale at the molecular level on why these improved conditions enhance XX retaining. Can the DE analysis suggest any pathways (other than GSK/MEK) or genes to be involved in the retention of the 2 X chromosomes? (although this does not affect the validity or the importance of this study).

Minor points:
1. For the general reader, it is not immediately clear what is different from the culturing conditions published in Mulas et al., vs. your paper. I think it would help the reader to highlight the main differences at a high level in the intro and/or in the discussion.
2. It would be nice to have some data from longer passaging in Mulas vs Keniry's vs 2i/LIF/serum conditions i.e. >p20 in the supplementary (if possible).
3. We have found that classic 2i conditions + 0,5-2% serum also stabilize the XX phenotype – have you tested this condition? We do not find any priming, at least in the context of XCI.
4. XX karyotype has been reported to be more stable in F1 hybrids such as cast/129. Do you see any major differences between pure BL/6 strains and F1 hybrids?
5. It would be good to compare your differentiation to other published data obtained in differentiating female XX to be added to Fig. 2, if it is possible to find comparable datasets. Ref 20/34 data comes from male cell lines only, I believe.

First revision

Author response to reviewers' comments

We would like to thank the Reviewers for their careful critique of our work and for their constructive comments. We have found the process to be genuinely helpful and believe it has resulted in a substantial improvement to the manuscript. The most significant additions are as follows:

- Evidence that high quality purified XX Xmas mESCs can be produced by fluorescence activated cell sorting and remain stable for 10 further passages. This opens the opportunity for continued FACS purification of XX cells over time.
- Testing lowered MEK inhibitor and the addition of xeno-free serum replacement on XX retention, although neither had an appreciable effect.
- A likely explanation as to why our method improves retention of XX cells, together with modelling to support this hypothesis.
- Additional analysis of our RRBS data, including a comparison to published data, showing that the XX Xmas cells behave as expected.
- A table detailing the points of difference between our method and the Mulas2019 method, including a rationale for each change.

Further changes and our point-by-point response to the Reviewers comments are detailed below, where their comments are in regular black font and our replies in blue italics. Changes to the text of the manuscript are indicated within the revised manuscript in red.
We hope in combination these changes are considered satisfactory; we believe they have greatly strengthened the paper.

© 2022. Published by The Company of Biologists under the terms of the Creative Commons Attribution License (https://creativecommons.org/licenses/by/4.0/).

We look forward to hearing from you.

Best wishes,
Andrew Keniry and Marnie Blewitt.

Reviewer 1 Advance Summary and Potential Significance to Field:
In this manuscript, Keniry et al. describe a new protocol to allow XX mouse ES cells to maintain their XX karyotype in a long-term culture compared to the 2i/LIF condition, which has been used as the best protocol to maintain naïve mouse ES cells. XX ES cells are known to easily lose one X chromosome during culture and become X0 karyotype. The development of an optimized culture condition to maintain XX karyotype is beneficial to investigate XCI as well as the pluripotency of female cells. By modifying the 2i/LIF culture condition, the paper shows XX ES cells can retain their XX karyotype as long as 10 days. These female ES cells also have a genome-wide CpG hypomethylation, another feature of the pluripotency of XX ESCs. The paper also shows that male ESCs can stably retain transcriptome and karyotype in this new culture condition.

Overall, the paper defines a novel culture condition that allows XX ES cells to be maintained in the naïve pluripotency for longer time than 2i/LIF. Female XX ES cells, however, retain XX karyotype until day 10 and then appear to start a rapid loss of one X chromosome with the new method. The XX karyotype is maintained about 2 times longer with the new method than the conventional 2i/LIF condition (10 days vs 5 days based on Fig. 1B), which would not be a strong improvement. In addition, the authors also describe that FACS sorting to select XX GFP/mCherry double-positive cells does not work effectively, narrowing the possibility to maintain XX karyotype. I, therefore, think that the current manuscript does not have much improvement and cannot be accepted for publication without adding more strong data.

Reviewer 1 Comments for the Author:
Major points:

At least three major points need to be addressed and added to the current manuscript.
1. It is critical to extend the duration of XX karyotype retention. The current data shows XX karyotypes can be maintained until day 10, which would not be long enough for several experiments such as creating a stable transgenic line. Therefore, I think XX karyotype should be maintained for a longer time.

We agree that any greater extension of the XX karyotype would be beneficial. It is important to note that our current method provides an incremental improvement towards maintenance of the XX karyotype, that enables significantly more complex experimentation to be performed. While we agree that the issue of X chromosome loss has not been entirely overcome, the incremental improvement will be of huge benefit to the community for this reason, as it has been for us.

The reviewer notes that as per our graphs the XX karyotype is only maintained for 10 days. While we have only tracked the XX state for 10 days in these experiments, the cells were grown from single blastocysts for 10-14 days before starting this experiment in order to have sufficient cells. So the real culture period is closer to 24 days. We have added these comments to the text on page 4 to ensure this is clear.

We now also include experiments designed to improve XX karyotype maintenance by simple modifications to the 2i+LIF media, including halving the amount of MEK inhibitor, adding 2% knockout serum replacement (KSR) and both of these in combination. Unfortunately, these measures did not improve karyotype maintenance, however will likely be beneficial knowledge for the community. These results are shown in the manuscript as Fig. 1G.

2. Alternatively, even if it is difficult to maintain XX karyotype for more than 10 days, a method to select XX cells from X0/XX mixed population would overcome this limitation. This point needs to be further examined.
We thank the reviewer for highlighting this point, especially as our reporters were set up to allow FACS. Upon reflection, our wording in the manuscript was negative compared with the reality. Previously, when we purified cells by FACS the rate of XX karyotype loss was accelerated, thereby

© 2022. Published by The Company of Biologists under the terms of the Creative Commons Attribution License (https://creativecommons.org/licenses/by/4.0/).

negating the benefit of sorting. We have now made some modifications to our sorting method, including sorting from a population of cells with a higher XX/XO ratio, adding KSR to the collection tube and sorting on a more modern instrument which is now available to us. These measures have greatly improved maintenance of the XX karyotype post-sort and we now provide data showing we can maintain the XX karyotype for 10 passages post sort, which we include in the manuscript as Fig. 1H. This possibility substantially increases the amount of XX daughter Xmas cells that can be produced from a derivation and therefore the range of experimentation possible is greatly increased. We thank the reviewer for suggesting we revisit this topic.

3. Do these ES cells cultured with the new method have more efficient germ line transmission? The efficiencies of germ line transmission with both male and female ES cells cultured with the new method should be examined. As male ES cells retain transcriptome and karyotype, this new method would also improve the germ line transmission of male ES cells than 2i/LIF condition. It is also important to examine the efficiency of germ line transmission of female ES cells to study the female pluripotency.

Improved germline transmission from our culture conditions would be of huge benefit and warrants testing. Indeed, there are myriad uses for mESCs that may warrant testing with our conditions, however we believe testing such applications is outside of the scope of this initial publication and would be better tested by the downstream experienced users together with their application specific requirements given the high cost and lengthy time required for such a study to be performed properly.

Specific points:
1. In Fig. 2C, the authors describe that the XX FVB/CAST transcriptome obtained with the new culture condition was more highly correlated with the one obtained with 2i/LIF culture condition than serum-containing medium. In Fig. 4A, however, they mention that male ES cells cultured with the new protocol and 2i/LIF have different PCA profiles. These descriptions are quite confusing. Is it possible for the authors to perform a Pearson correlation analysis for male ES cells and PCA for female ES cells in both the new culture condition and 2i/LIF?

The reason for this disparity is because Fig 2C compares several disparate culture methods, while Fig 4A compares specifically the Keniry2022 method with Mulas2019, which are more closely aligned. We have performed the additional Pearson correlation on male mESCs and provide this as Fig. S2A. The Pearson correlation shows an equivalent result to the PCA.

2. They show that female ES cells have longer doubling time, 26.5 hr with the new culture condition and 43.4 hr with 2i/LIF. They also describe that male ES cells with 2I/LIF grow more rapidly (12 – 30 hr). Is this shorter doubling time specific for female ES cells? How fast do male ES cells grow in the new culture condition?

We have now produced growth curves for male mESCs in our culture conditions and include these as Fig. 4A. These new data demonstrate that doubling time for male mESCs is also decreased, however the benefit is not as great as was observed for females. Whereas females were improved from 43.4 to 26.5h, males only went from 24.5 to 21.8h. Therefore the benefit in doubling time is much more pronounced in females than in males.

3. In Fig. 2A, the authors perform DNA FISH with Huwe1 probe and show that the majority of XX ES cells retain two Huwe1 spots. However, two Huwe1 spots could also represent X0 tetraploid karyotype? To avoid this possibility, it is necessary to perform flowcytometry or DNA FISH with an autosomal probe to prove that they are diploid, but not tetraploid.

It is a formal possibility that XO cells that are tetraploid (XXOO cells) are the reason for why we observe 2 Huwe1 foci by DNA FISH. However, this it is highly unlikely when considering our current data. Most importantly, given that it would be likely that cells could become tetraploid then lose an X (or two) or lose an X then become tetraploid, if tetraploidy were an issue we should see a much more heterogeneous population of cells, including cells with 4 Huwe1 foci (XXXX), cells with 2 Huwe1 foci (XXOO or XX) and cells with 1 Huwe1 focus (XO). Over our timecourse we never observe 4 Huwe1 foci, suggesting that tetraploidy is not occurring. Furthermore, to our knowledge tetraploidy has not been reported as an issue in mESC culture.

© 2022. Published by The Company of Biologists under the terms of the Creative Commons Attribution License (https://creativecommons.org/licenses/by/4.0/).

Given that these cells are freshly derived from blastocysts, we consider it highly unlikely that tetraploidy would have occurred so rapidly and at such high penetrance in all our replicate samples so early after derivation. To formally exclude tetraploidy, we would need to rederive FVB/Cast mESC and perform either flow cytometry analyses of ploidy or large amounts of DNA FISH. Given that we do not freeze our mESCs this would be a significant undertaking given the highly unlikely occurrence of tetraploidy in these cells and so we have not investigated further.

4. They describe that lower concentration of MEK inhibitor does not solve the problem entirely. The authors should clearly mention which problem they were not able to solve.

As discussed above, we now provide data for this experiment. While we have previously observed a benefit of using low Mek media, we did not see such an effect in this set of experiments, suggesting that the benefits may be cell line specific. As the new data has far more replicates than our initial experiment, we include the negative data in the manuscript and have removed the original observation from the text. We hope this will be helpful for the field.

5. Although it is briefly described in the text, it is still difficult to understand which points the authors have modified from the 2i/LIF protocol. I think the authors should present a table that shows the differences between the new protocol and the prior 2i/LIF condition. In addition, it is helpful to show the pictures of ES cells cultured in the new condition. Many researchers may not be familiar with suspension culture of ES cells and need to see how the cells look like.

Thank you for this suggestion, a table is an excellent way to demonstrate the points of difference within our method. We now provide this table in the manuscript as Table 1. Additionally, we provide brightfield images of cells cultured with our method and the Mulas2019 method in the manuscript as Fig 1B.

6. In Fig. 3, the authors show that XX ES cells grown with the new method retain hypomethylated CpG in repeat elements. The passage number of ES cells used in this analysis needs to be indicated.

These cells were passage 12. We've added this information to the Methods section on page 12 of the manuscript and to the legend for Fig. 3.

Reviewer 2 Advance Summary and Potential Significance to Field:
In this manuscript the authors introduce a new method to culture mouse ESCs which stabilizes against the loss of an X chromosome in XX females. They further characterize the genome, transcriptome and methylome of cells grown in their culture conditions and previously published conditions, demonstrating that their culture conditions more closely resemble freshly derived ESC cultures. We think that this will be a valuable contribution to the field, as X chromosome loss is very problematic for studying X chromosome inactivation and X chromosome content should be considered in all studies to ensure results are relevant to both males and females. See comments below for how we believe this manuscript could be improved:

Reviewer 2 Comments for the Author:
For your RRBS, you only compare your XX and XO populations. Could you compare these to a male or freshly derived female RRBS sample to demonstrate that the hypomethylation seen in your XX cells is proper and that it is the XO cells with problematic DNA methylation.

Thank you for this suggestion to provide a direct comparison to existing datasets. We have now performed a reanalysis of RRBS data for XX and XY mESCs, published in Choi et. al, 2017 Cell Stem Cell, and compare it to our data, finding a very high correlation. These data suggest that the hypomethylation observed in our XX mESCs is in the correct locations. This analysis is in the manuscript as Fig. 3D.

In your RRBS, could you separately compare X chromosome DNA methylation and autosomal DNA methylation, to see if X chromosome differences may be driving the hypomethylation differences you observe.

© 2022. Published by The Company of Biologists under the terms of the Creative Commons Attribution License (https://creativecommons.org/licenses/by/4.0/).

We now provide plots showing X chromosome and autosome methylation separately as Fig.3B and 3C. The hypomethylation is global, as reported previously for female mESCs.

2nd paragraph page 6, I think there is a typo saying Keniry2019. I think it should instead be Keniry 2022 or Mulas 2019

Corrected, thank you.

I suggest the addition of a small table demonstrating the difference between your culture conditions and those of Mulas2019, as this may provide an easily digestible summary of what you have changed.

Thank you for this suggestion, a table is an excellent way to demonstrate the points of difference within our method. We now provide this table in the manuscript as Table 1.

Figure 2B: Please provide the number of cells counted per condition. In the protocol I did not understand this sentence “Note, when passaging low numbers of cells the last drop should be blotted with a tissue to avoid significantly diluting the 2i media.“

We have added the cell numbers to Fig. 2B and rewritten the admittedly confusing sentence.

Can you provide the brand and catalogue number of the “round wells of non-tissue culture treated plates "

We have added these details.

Can you discuss the doubling times of Song et al Stem Cell Reports 2019?

This was an oversight. We have added a brief discussion of this study on page 8.

Can you describe better, more specifically, the new changes to the protocol in the main manuscript on page 4?

We have described the more major changes to the protocol in the main body of the text, however for brevity we have opted to show the many minor alterations in the suggested table.

Reviewer 3 Advance Summary and Potential Significance to Field:
Keniry et al. using their recently-derived double-reporter female cell line and other mouse ES lines, report the set-up of a new protocol to stabilize the XX genotype in mouse female ESCs during culturing. Working with female ESCs is hard, and this protocol might bring some relief/help to people working in X Chromosome Inactivation (XCI). It might also encourage more widespread use of female ESCs in research. Therefore I think that this work is important. Not just for the XCI field but for the whole stem cell community. The manuscript, however, can be further improved. If my criticisms are properly addressed, this work can be accepted for publication.

Reviewer 3 Comments for the Author:
Major criticism:

1. Considering how many variables can be optimized in such types of protocol, it is not clear - at all - how you arrived at the final version of your improved protocol. What was the primary rationale for setting up different culturing conditions or handling procedures such as splitting? How many conditions were tested? Can you exclude that the genetics/epigenetics of the cell lines used in this work contributed to the observed phenotype?

Our method was determined empirically, through hundreds of rounds of derivations and lines subsequently going XO. The majority of the changes we made have not been tested in direct comparisons- bar those that we show.

As suggested below, we now include a table that highlights all points of difference between our protocol and the Mulas2019 protocol, along with a rationale for each change. This is Table 1 in

© 2022. Published by The Company of Biologists under the terms of the Creative Commons Attribution License (https://creativecommons.org/licenses/by/4.0/).

the manuscript. We believe this provides a more easily interpretable explanation of the changes for readers.

We have now also directly tested the addition of 2% knockout serum replacement and low concentration of Mek inhibitor, however neither of these measures in isolation or together had a stabilising effect on karyotype. These data are in the manuscript as Fig. 1G.

1. There is a lack of rationale at the molecular level on why these improved conditions enhance XX retaining. Can the DE analysis suggest any pathways (other than GSK/MEK) or genes to be involved in the retention of the 2 X chromosomes? (although this does not affect the validity or the importance of this study).

We have performed the pathway analysis as suggested, however the results highlight mitochondrial and ribosomal pathways, similarly to our GO analysis (Fig. 4C). We do think we now have a reasonable explanation for why XX mESCs are retained in our system though. In another set of experiments for these revisions we have determined the growth rate of male mESCs and find that similarly to females the rate is faster by our method than the Mulas2019 method, however the improvement is far greater in female mESCs (a drop from 43.4 to 26.5h in females, whereas a drop from 24.5 to 21.8h in males). This suggests that the growth advantage that our method offers is greater for XX cells rather than for XO or XY cells. Therefore, XO cells take longer to outcompete the XX cells under our conditions. We have modelled this scenario using the doubling times we determined experimentally and find that this explanation is more than able to account for the improved XX retention we observe with our method. This is discussed on page 8 of the manuscript and the male growth curves and modelling appear as Fig. 4A and Fig. 4G respectively.

Minor points:

1. For the general reader, it is not immediately clear what is different from the culturing conditions published in Mulas et al., vs. your paper. I think it would help the reader to highlight the main differences at a high level in the intro and/or in the discussion.

As mentioned above, we now summarise these details as Table 1 in the manuscript which we do believe will help the reader.

2. It would be nice to have some data from longer passaging in Mulas vs Keniry's vs 2i/LIF/serum conditions i.e. >p20 in the supplementary (if possible).

It is only useful to continue until the XX karyotype is lost. This is approximately 10 passages from when we begin the assay, however it takes 4-6 passages to bulk the cells up to sufficient numbers to begin the assay. So the data presented actually represents passage 14-16. We have discussed this on page 4 of the manuscript.

We now provide additional data demonstrating that XX Xmas mESCs can be purified by FACs and remain stable for approximately 10 further passages. These data are Fig. 1H in the manuscript and represent cells that have been passaged in excess of 20 times and excitingly suggest that purification by FACs may be a solution for long term maintenance of XX Xmas mESCs.

3. We have found that classic 2i conditions + 0,5-2% serum also stabilize the XX phenotype – have you tested this condition? We do not find any priming, at least in the context of XCI.

Thank you for this suggestion. We were keen to keep our media defined, so opted to test the addition of 2% xeno-free serum replacement. As mentioned above, this did not have a stabilising effect on XX karyotype.

4. XX karyotype has been reported to be more stable in F1 hybrids such as cast/129. Do you see any major differences between pure BL/6 strains and F1 hybrids?

Yes, we find that F1 hybrids tend to maintain their XX karyotype longer than our Bl/6 Xmas cells. However, they still go XO, as shown in Figure 2a.

© 2022. Published by The Company of Biologists under the terms of the Creative Commons Attribution License (https://creativecommons.org/licenses/by/4.0/).

5. It would be good to compare your differentiation to other published data obtained in differentiating female XX to be added to Fig. 2, if it is possible to find comparable datasets. Ref 20/34 data comes from male cell lines only, I believe.

We agree, this would be nice to perform, however we're not aware of a suitable dataset of female mESC differentiation using comparable differentiation conditions (undirected differentiation) and native Xist expression (other labs direct Xist with Dox induction). Therefore, direct comparisons would be challenging to interpret appropriately and so not helpful at this stage.

Second decision letter

MS ID#: DEVELOP/2022/200845

MS TITLE: A method for stabilising the XX karyotype in female mESC cultures

AUTHORS: Andrew Keniry, Natasha Jansz, Peter F Hickey, Kelsey Breslin, Megan Iminitoff, Tamara Beck, Quentin Gouil, Matthew E Ritchie, and Marnie E Blewitt

I have now received all the referees reports on the above manuscript, and have reached a decision. The referees' comments are appended below, or you can access them online: please go to BenchPress and click on the 'Manuscripts with Decisions' queue in the Author Area.

We are about to accept your manuscript, but reviewers raised small concerns that I would like to clarify. Please address these small points maybe editorially in your revised manuscript and detail them in your point-by-point response. If you do not agree with any of their criticisms or suggestions explain clearly why this is so. If it would be helpful, you are welcome to contact us to discuss your revision in greater detail. Please send us a point-by-point response indicating your plans for addressing the referee's comments, and we will look over this and provide further guidance.

Reviewer 1

Advance summary and potential significance to field

I would like to thank the authors for addressing my concerns. The paper is much stronger with new data and re-writing. I think the explanation that less stress with the new protocol preserves XX karyotype and the shorter doubling time is reasonable. This new protocol benefits many researchers working on female ES cells and could be fundamental for future improvement of the protocol.

Comments for the author

One typo on page 5, C57/Bl6.

Reviewer 2

Advance summary and potential significance to field

See previous review

Comments for the author

The authors have answered most of our comments to our satisfaction however I am slightly worried by a response to reviewer 1. In figure 1, you mention that the cells were cultured for 10-14 days prior to the start of your experiments. Was the amount of time cells were grown before the start of the experiment matched between the two experimental conditions?

© 2022. Published by The Company of Biologists under the terms of the Creative Commons Attribution License (https://creativecommons.org/licenses/by/4.0/).

What I am worried about is if there is a difference between the cells that were cultured for 10 days prior to the experiment and those cultured for 14 days prior to the experiment, especially if the time to grow cells prior to your experiment differed between your experimental conditions. If you kept track of the time cells were cultured prior to the experiments, it would be useful, to note the time starting from the beginning of culture instead of when you started measuring. This minor point can probably we dealt with with textual changes.
Congratulations to the authors on this interesting story!

Reviewer 3

Advance summary and potential significance to field

The authors satisfactorily addressed all my concerns; thank you. One outstanding comment is that I also want to see the karyotype of these cells stable for several weeks after ES derivation rather than 1-2 weeks.

Comments for the author

NA

Second revision

Author response to reviewers' comments

Reviewer 1 Advance Summary and Potential Significance to Field:
I would like to thank the authors for addressing my concerns. The paper is much stronger with new data and re-writing. I think the explanation that less stress with the new protocol preserves XX karyotype and the shorter doubling time is reasonable. This new protocol benefits many researchers working on female ES cells and could be fundamental for future improvement of the protocol.
Reviewer 1 Comments for the Author:
One typo on page 5, C57/Bl6.

Amended, thank you.

Reviewer 2 Advance Summary and Potential Significance to Field:
See previous review

Reviewer 2 Comments for the Author:
The authors have answered most of our comments to our satisfaction however I am slightly worried by a response to reviewer 1. In figure 1, you mention that the cells were cultured for 10-14 days prior to the start of your experiments. Was the amount of time cells were grown before the start of the experiment matched between the two experimental conditions?

What I am worried about is if there is a difference between the cells that were cultured for 10 days prior to the experiment and those cultured for 14 days prior to the experiment, especially if the time to grow cells prior to your experiment differed between your experimental conditions. If you kept track of the time cells were cultured prior to the experiments, it would be useful, to note the time starting from the beginning of culture instead of when you started measuring. This minor point can probably we dealt with with textual changes.
Congratulations to the authors on this interesting story!

Each replicate was performed from a matched Xmas blastocyst derivation, split between culture conditions on the day of the experimental timecourse. Therefore, each replicate is exactly time matched. This wasn't clear, so we have added the following text to the methods section:

© 2022. Published by The Company of Biologists under the terms of the Creative Commons Attribution License (https://creativecommons.org/licenses/by/4.0/).

"For comparison of our method with the Mulas2019 method, Xmas mESC lines were derived over approximately 10 to 14 days. At the beginning of our experimental timecourse each line was split into both culture conditions, such that each replicate has a matched sample in each condition."

Reviewer 3 Advance Summary and Potential Significance to Field:
The authors satisfactorily addressed all my concerns; thank you. One outstanding comment is that I also want to see the karyotype of these cells stable for several weeks after ES derivation rather than 1-2 weeks.

Each experiment was derived in culture for 10 to 14 days prior to our 10-day timecourse, such that we have measured karyotype stability for much longer than 1-2 weeks. This was already mentioned in the text, but we hope that the methods amendment detailed above makes this point clearer.

Reviewer 3 Comments for the Author:

NA

Third decision letter

MS ID#: DEVELOP/2022/200845

MS TITLE: A method for stabilising the XX karyotype in female mESC cultures

AUTHORS: Andrew Keniry, Natasha Jansz, Peter F Hickey, Kelsey Breslin, Megan Iminitoff, Tamara Beck, Quentin Gouil, Matthew E Ritchie, and Marnie E Blewitt
ARTICLE TYPE: Techniques and Resources Report

I am happy to tell you that your manuscript has been accepted for publication in Development, pending our standard ethics checks.

© 2022. Published by The Company of Biologists under the terms of the Creative Commons Attribution License (https://creativecommons.org/licenses/by/4.0/).
